# Fungal Endophyte Communities of Three Agricultural Important Grass Species Differ in Their Response Towards Management Regimes

**DOI:** 10.3390/microorganisms7020037

**Published:** 2019-01-27

**Authors:** Bernd Wemheuer, Torsten Thomas, Franziska Wemheuer

**Affiliations:** 1 Genomic and Applied Microbiology and Göttingen Genomics Laboratory, Institute of Microbiology and Genetics, Georg-August University of Göttingen, D-37077 Göttingen, Germany; bwemheu@gwdg.de; 2 Centre for Marine Bio-Innovation and School of Biological, Earth and Environmental Sciences, University of New South Wales, Sydney, NSW 2052, Australia; t.thomas@unsw.edu.au; 3 Division of Agricultural Entomology, Department of Crop Sciences, Georg-August University of Göttingen, D-37077 Göttingen, Germany

**Keywords:** Fungal endophytes, associated fungi, grassland management, putative fungal life strategies, high-throughput sequencing, agriculturally important grass species, fertilization, mowing frequency

## Abstract

Despite the importance of endophytic fungi for plant health, it remains unclear how these fungi are influenced by grassland management practices. Here, we investigated the effect of fertilizer application and mowing frequency on fungal endophyte communities and their life strategies in aerial tissues of three agriculturally important grass species (*Dactylis glomerata* L., *Festuca rubra* L. and *Lolium perenne* L.) over two consecutive years. Our results showed that the management practices influenced fungal communities in the plant holobiont, but observed effects differed between grass species and sampling year. Phylogenetic diversity of fungal endophytes in *D. glomerata* was significantly affected by mowing frequency in 2010, whereas fertilizer application and the interaction of fertilization with mowing frequency had a significant impact on community composition of *L. perenne* in 2010 and 2011, respectively. Taken together, our research provides a basis for future studies on responses of fungal endophytes towards management practices. To the best of our knowledge, this is the first study simultaneously assessing fungal endophyte communities in aerial parts of three agriculturally important grass species over two consecutive years.

## 1. Introduction

Endophytic fungi have been found in every plant species investigated so far [1,2,3]. In recent years, the definition of “endophyte” has been changed several times (reviewed in [1,4]). According to Hardoim et al. [4] and Porras-Alfaro and Bayman [5], the term “endophyte” should refer to the habitat but not to the function or the nature of the relationship with the plant. In contrast, Le Cocq et al. [6] and van Overbeck and Saikkonen [7] suggested that endophytes are those ‘microbes which colonize internal plant tissues for at least parts of their life cycle without causing disease symptoms under any known circumstances’. In the present study, we adopt the concept of Hardoim et al. [4] and Porras-Alfaro and Bayman [5], which allows to include latent pathogens, latent saprotrophs and non-pathogenic endophytes.

Several endophytic fungi play important roles in plant health by promoting plant growth and providing resistance to multiple stresses, such as drought or nutrient deficiency [1,8,9]. Some endophytic fungi are able to produce compounds that have growth-inhibitory activities toward herbivores and phytopathogens and thus can be used as biological control agents [2,10]. Given the important ecological and economic role of endophytic fungi in a sustainable agriculture [3,6,11], it is crucial to decipher compositional and functional responses of these fungi towards agricultural practices. 

Recent research has shown how management can influence foliar fungal assemblages. For instance, Pancher et al. [12] showed that fungal endophyte communities in grapevines from organically managed farms were different from those with integrated pest management. Similarly, differences between communities in relation to management at various disturbance levels ranging from undisturbed sites with light sheep grazing over intermediate (overgrazed) to disturbed sites (reseeded ryegrass lay) were observed in a study on culturable root-endophytic fungal communities in temperate grasslands [13]. By contrast, foliar fungal assemblages in *Lolium perenne* L. were not affected by land-use intensity (i.e., different levels of mowing, grazing and fertilization), though these assemblages did differ among study regions and season [14].

Seghers et al. [15] found no effect of fertilizer application on the endophytic community in roots of *Zea mays* L. using denaturing gel gradient electrophoresis (DGGE). On the contrary, nitrogen addition had an impact on the overall community composition of fungi associated with shoots of three boreal moss species [16]. As most studies have focused on (culturable) root endophytes [14,15] or fungi belonging to the clavicipitaceous endophytes [17,18,19], it is still unknown how different management regimes affect the entire fungal endophyte community in plants, and in particular in the aerial parts of different grass species.

In two previous studies, the effect of grassland management practices on bacterial endophyte communities in aerial parts of three grass species (*Dactylis glomerata* L., *Festuca rubra* L. and *L. perenne*) over two consecutive years were investigated by DGGE and pyrotag sequencing [20,21]. These grass species were chosen because they have a high agronomic importance and because they differ in their habitat preferences [22]. Both *D. glomerata* and *L. perenne* have a higher tolerance against mowing compared to *F. rubra*, while *L. perenne* has a higher tolerance against higher nitrogen supply than the other two species [22]. The two previous studies showed that mowing frequencies in combination with fertilizer application had a significant impact on the community composition of bacterial endophytes, but this was dependent on the grass species and the sampling year [20,21]. In another study conducted on the same experimental field, the impact of mowing frequency and fertilizer application on *Neotyphodium* species was investigated [17]. Here, fertilizer application increased the level of fungal colonization, while mowing had no effect. These results suggest that commonly employed management practices could affect foliar fungal endophyte communities as well.

In the present study, we used Illumina-based sequencing of the internal transcribed spacer region 2 (ITS2) region to assess the effect of management practices (i.e., fertilizer application and mowing frequency) on fungal endophyte communities in aerial parts of *D. glomerata*., *F. rubra* and *L. perenne*. To better understand plant-fungal interactions regarding grass species and management practices, correlation-based indicator species analyses were performed. In addition, putative life strategies (i.e., trophic modes) of the endophytic fungi found were determined using the FUNGuild database [23]. This is of particular interest as differences in structure and in the putative life strategies of the entire fungal endophyte community in three grass species among different management practices have not been investigated so far.

We focused on three main hypotheses: (i) fungal endophyte diversity and community composition differ among the investigated grass species and between the sampling years, (ii) fungal endophyte communities in the grass species respond differently towards management practices and (iii) the putative life strategies of fungal endophytes are associated with grass species and management practices. To our knowledge, this is the first study simultaneously assessing fungal endophyte communities in aerial parts of three agriculturally important grass species over two consecutive years.

## 2. Materials and Methods 

### 2.1. Study Site and Experimental Design

The Grassland Management Experiment (GrassMan) was a long-term field experiment to study the effect of grassland management practices and intensity on biodiversity. It was established in spring 2008 at a semi-natural, moderately species-rich, temperate grassland site at the Relliehausen Experimental Farm in the Solling Mountains in Lower Saxony, Germany (51°44′53′′ N, 9°32′43′′ E, 490 m a.s.l.). The full-factorial design of GrassMan included three levels of plant species richness (dicot-reduced, monocot-reduced and species-rich plots) as well as two management practices. The plant species richness gradient was achieved by selective herbicide application which either reduced dicot or monocot species diversity (see Petersen et al. [24]). One third of the plots were left untreated as control (species-rich). The application of herbicides took place on 31 July 2008 and resulted in significant changes in plant species richness and functional group abundances [24]. 

Management practices for the three plant species richness levels included two mowing frequencies (once per year in July vs three times per year in May, July and September) and two fertilization treatments (fertilizer application with nitrogen/phosphorous/potassium (NPK) vs no fertilizer application). The combination of these management practices resulted in four different management levels: (1) no fertilizer application and mown once (NPK- 1x), (2) no fertilizer application and mown three times (NPK- 3x), (3) fertilizer application and mown once (NPK+ 1x), and (4) fertilizer application and mown three times (NPK+ 3x). Each treatment was replicated six times on 15 × 15 m plots, resulting in 72 plots. The plots were arranged in a Latin Square design with six rows and six blocks. Please see Petersen et al. [24] for a detailed description of the experimental design.

Plant samples were taken from the GrassMan experimental field as described previously [20]. In brief, aerial parts (leaves and part of the tillers) of *L. perenne*, *F. rubra* and *D. glomerata* were randomly collected from each management level and grass species from dicot-reduced plots on 19 September 2010 and the 12 September 2011. Only plants from dicot-reduced plots were sampled due to the higher abundances of the target grass species on these plots. The plants were collected with at least 1 m distance from each other. Ten plants per grass species and plot were sampled, with one exception: *L. perenne* samples were collected only from two non-fertilized and three fertilized plots mown once in 2010 due to the absence of this grass species in the other plots. Only plants without obvious disease symptoms, such as chlorosis, leaf spots or pathogen-induced lesions, were collected. Plant samples were immediately cooled down to 4 °C and transferred to the laboratory. During the study period, precipitation and mean temperature were 93.6 mm and 11.42 °C in September 2010 and 54.75 mm and 14.75 °C in September 2011, respectively.

### 2.2. Surface Sterilization and Extraction of Total Community DNA

Surface sterilization of collected aerial plant material was performed as described in Wemheuer et al. [20]. To confirm the success of the sterilization, 100 *μ*l aliquots of the water used in the final washing step were plated on common laboratory media plates. The plates were incubated in the dark at 25 °C for at least 2 weeks. No growth of microorganisms was observed. As a second control, water from the final washing step was subjected to polymerase chain reaction (PCR) targeting the ITS region. No amplification was detected. The surface-sterilized plant material was ground to a fine powder in liquid nitrogen using an autoclaved mortar and pestle. Aliquots of the ground tissue were subsequently stored at −20 °C until DNA extraction. DNA was extracted employing the peqGOLD Plant DNA Mini kit (Peqlab, Erlangen, Germany; now VWR) according to the manufacturer’s instructions with two modifications as described previously [20]. The modifications included a beat-beating step and the addition of proteinase K.

### 2.3. Amplification of the Fungal ITS Region

Fungal communities in aerial plant parts were assessed by nested PCR targeting the ITS region using the same template DNA as in two previous studies investigating bacterial endophytes [20,21]. The PCR was performed as described previously [25,26]. In the first PCR, the primers ITS1-F_KYO2 (5′-TAGAGGAAGTAAAAGTCGTAA-3′) [27] and ITS4 (5′- TCCTCCGCTTATTGATATGC-3′) [28] were used to suppress co-amplification of plant-derived ITS regions. The ITS2 region was subsequently amplified as described for the first PCR using approximately 50 ng product of the first PCR and the primers ITS3_KYO2 [27] and ITS4 [28] containing the MiSeq adaptors (underlined): MiSeq-ITS3_KYO2 (5′-TCGTCGGCAGCGTCAGATGTGTATAAGAGACAGGATGAAGAACGYAGYRAA-3′) and MiSeq-ITS4 (5′-GTCTCGTGGGCTCGGAGATGTGTATAAGAGACAGTCCTCCGCTTATTGATATGC-3′). Negative controls in both PCRs were performed using the PCR reaction mixture without template.

The resulting PCR products per sample were checked for appropriate size, purified using the peqGOLD Gel Extraction kit (Peqlab) and pooled in equal amounts. Quantification of the purified PCR products was performed using the Quant-iT dsDNA HS assay kit and a Qubit fluorometer (Thermo Scientific) as recommended by the manufacturer. The best three of the six replicates with the strongest specific PCR signal and highest DNA concentrations were chosen for sequencing with one exception: as described above, we had only two *L. perenne* samples from non-fertilized plots mown once in 2010. In total, 71 (2010: 35; 2011: 36) samples were analyzed in this study (Table S1). Quantified PCR products were barcoded using a Nextera XT-Index kit (Illumina, San Diego, CA, USA) and the Kapa HIFI Hot Start polymerase (Kapa Biosystems, Wilmington, MA, USA). The Göttingen Genomics Laboratory sequenced the ITS2 region employing the MiSeq Sequencing platform and the MiSeq Reagent Kit v3 (2 × 300 cycles) as recommended by the manufacturer (Illumina). All fungal samples were sequenced on the same MiSeq run.

### 2.4. Processing of ITS Datasets

Sequencing data were initially quality filtered with the Trimmomatic tool version 0.36 [29]. Low-quality reads were truncated if the quality dropped below 15 in a sliding window of 4 bp. Subsequently, all reads shorter than 100 bp and orphan reads were removed. Remaining sequences were merged, quality-filtered and further processed with USEARCH version 10.0.240 [30]. Filtering included the removal of reads shorter than 250 bp or longer than 450 bp as well as the removal of low-quality reads (expected error >1) and reads with more than one ambiguous base. Files of the processed sequences for each sample were concatenated to one file and subsequently dereplicated into unique sequences. These sequences were denoised and clustered in zero-radius operational taxonomic units (zOTUs; also called “exact sequence variants, see [31]) with the unoise3 algorithm implemented in USEARCH [30]. Chimeric sequences were removed *denovo* using the UCHIME algorithm during clustering [32]. Subsequently, remaining chimeric sequences were removed using UCHIME2 [32] in reference mode with the uchime release of the UNITE database version 7.2 [33]. 

To assign taxonomy, unique and chimera-free sequences were classified by BLAST alignment [34] against the UNITE+INSD database version 7.2 (released 2017-12-01) [33] with an e-value threshold of 1e-20. All non-fungal zOTUs were removed. All sequence reads were mapped on the final set of unique sequences to calculate the presence and abundance of each zOTU in each sample. The final zOTU table and sequence characteristics are provided as Tables S2 and S3, respectively. In order to calculate UniFrac distances and Faith’s phylogenetic diversity (Faith’s PD), zOTU sequences were aligned with MUSCLE v3.8.425 [35] and a phylogenetic tree was generated using RAxML version 8.2.4 [36]. 

### 2.5. Diversity and Statistical Analyses

All analyses were conducted in R version 3.5.0 [37]. Differences were considered as statistically and marginally significant with *P* ≤ 0.05 and *P* ≤ 0.1, respectively. Differences in sequencing depth among management practices, sampling year and grass species were tested using a Kruskal Wallis test. There were no significant differences in library size; and thus, data were not normalized prior to further analysis (with exception of the alpha diversity analysis). OTU tables were rarefied to 5741 fungal sequences per sample prior to alpha diversity analysis using the *rrarefy* function in *vegan* 2.5-2 [38]. Alpha diversity indices (richness, Shannon index of diversity, Faith’s phylogenetic diversity (PD), Chao1 and Michaelis-Menten Fit) were calculated using the R-packages *vegan* [38], *picante* version 1.7 [39] and *drc* version 3.0-1 [40]. To calculate the Michaelis-Menten Fit, rarefaction curves were generated using the *rarecurve* function in *vegan* [38]. The Michaelis-Menten Fit was subsequently calculated from the rarefaction curves using the *MM2* model within the *drc* package [40]. All alpha diversity indices were calculated 10 times per sample. The average of all iterations was used for further statistical analyses. The final table containing fungal richness and diversity is provided as Table S3. 

The impact of grass species and sampling year on fungal richness and diversity was tested using a repeated measures analysis of variance (ANOVA) with plot as error term [41]. The influence of fertilizer application and mowing frequency on fungal richness and diversity was subsequently tested for each individual grass species and sampling year separately. Alpha diversity data were tested for normal distribution using the Shapiro-Wilk’s test (*shapiro* function) and for homogeneity of variance using a Levene test (*leveneTest* function) via the R package *car* version 3.0-0 [42]. Because the distribution of fungal diversity, phylogenetic diversity and richness significantly differed from a normal distribution, differences in fungal alpha diversity measures were tested by Kruskal-Wallis test.

Potential differences in community structure were investigated by permutational multivariate analysis of variance (PERMANOVA) with 1000 random permutations using the *vegdist* and *adonis* function within the *vegan* package [38]. Four different dissimilarity measures were tested: Bray-Curtis, Jaccard, weighted as well as unweighted UniFrac. As Bray-Curtis and weighted UniFrac dissimilarities displayed a higher environmental sensitivity based on the higher coefficients of determination, only results for these distance measures are shown. Global differences (i.e., all samples) with regard to sampling year and grass species were constrained within each plot using the “strata” argument to account for nestedness in the experimental design. Subsequently, samples taken in 2010 and 2011 were analysed separately. Differences in community structure were visualized using the *metaMDS* function in the *vegan* package [38]. In case of significant *p*-values in PERMANOVA, we tested for differences in group homogeneity using permutational analysis of multivariate dispersions (PERMDISP) with 999 permutations using the function *betadisper* in the R package *vegan* [38], followed by a Tukey test with *P* value adjustment for multiple comparison. Differences in community composition between the management intensities and between the grass species were tested using pairwise PERMANOVA (available online: https://github.com/bwemheu/pairwise.adonis; version 0.1.0).

To identify zOTUs that are significantly associated with each grass species and the four management levels, multipattern analyses were applied with *multipatt* function from the *indicspecies* package [43]. The *multipatt* function analyses the association between species patterns and combinations of groups of sites, which provides an extra flexibility to qualitatively model the habitat preferences of the species of interest [44]. The biserial coefficients (*R*) with a particular management practice or grass species were corrected for unequal sample size using the function *r.g* [45]. The point-biserial correlation coefficient is a Pearson correlation computed between a quantitative vector (i.e., the vector containing the species abundance values at the various sites) and a binary vector (i.e., the vector of site membership values [43]. According to De Cáceres and Legendre [43], the point-biserial correlation coefficients are the better choice for species abundance data. 

To enhance reliability of the indicator analysis, only fungal zOTUs found in at least three samples were considered. All zOTUs present in all samples of a particular grass species were defined as core microbiome. In addition, we identified zOTUs found in all 71 samples. The core community and the fungal zOTUs associated with each grass species as well as with management practices and grass species were visualized using *Cytoscape* version 3.6.1 [46]. The results of the indicator analysis with regard to grass species combined with management practices are provided as Table S4. Differences in the relative abundance of predominant fungal genera (> 0.5% abundance in the entire dataset) were tested by pairwise *t*-test with *P* value adjustment (Benjamini-Hochberg) for multiple comparisons. 

### 2.6. Functional Prediction

Functional information was assigned to fungal zOTUs using FUNGuild [23]. Samples taken in 2010 and 2011 were analysed separately (Table S5). We kept guild assignment only to those zOTUs that were assignment with the confidence ranking of “probable” and “highly probable” as recommend [23]. The number of zOTUs assigned into guilds were plotted as relative percentages (number of zOTUs assigned to a specific guild divided by the number of all assigned zOTUs, called sequence richness). In addition, the zOTU richness was determined (the proportion of zOTUs assigned to a specific trophic mode). Differences in the sequence and zOTU richness were tested by pairwise *t*-test with Benjamini-Hochberg correction for multiple testing.

### 2.7. Nucleotide Sequence Accession Numbers

Sequence data are deposited in the Sequence Read Archive (SRA) of the National Center for Biotechnology Information (NCBI) under the accession number SRA750152.

## 3. Results

### 3.1. Fungal Endophyte Communities Are Dominated by Ascomycota and Basidiomycota

We collected aerial plant parts of *D. glomerata*, *F. rubra* and *L perenne* from the GrassMan Experimental Field over two consecutive years. Diversity and composition of fungal endophyte communities in 71 grass samples were assessed by Illumina-based sequencing targeting the fungal ITS2 region (Table S1). After quality filtering, denoising, and removal of potential chimeras as well as non-fungal sequences, a total of 4,601,322 high-quality sequences were obtained for further analysis. Sequences were assigned to 3230 fungal zOTUs (Table S2). Sequence numbers per sample varied between 6065 to 193,783, with an average of 64,807 sequences per sample (Table S3). Rarefaction curves and Michaelis-Menten Fits indicated that a major part of the fungal diversity (coverage: 76.4%) was recovered by the surveying effort (Figure S1, Table S3). A species accumulation curve further indicated that 96.3% of all fungal zOTUs (maximal number of zOTUs calculated = 3354) were recovered (Figure S1). 

**Figure 1:**
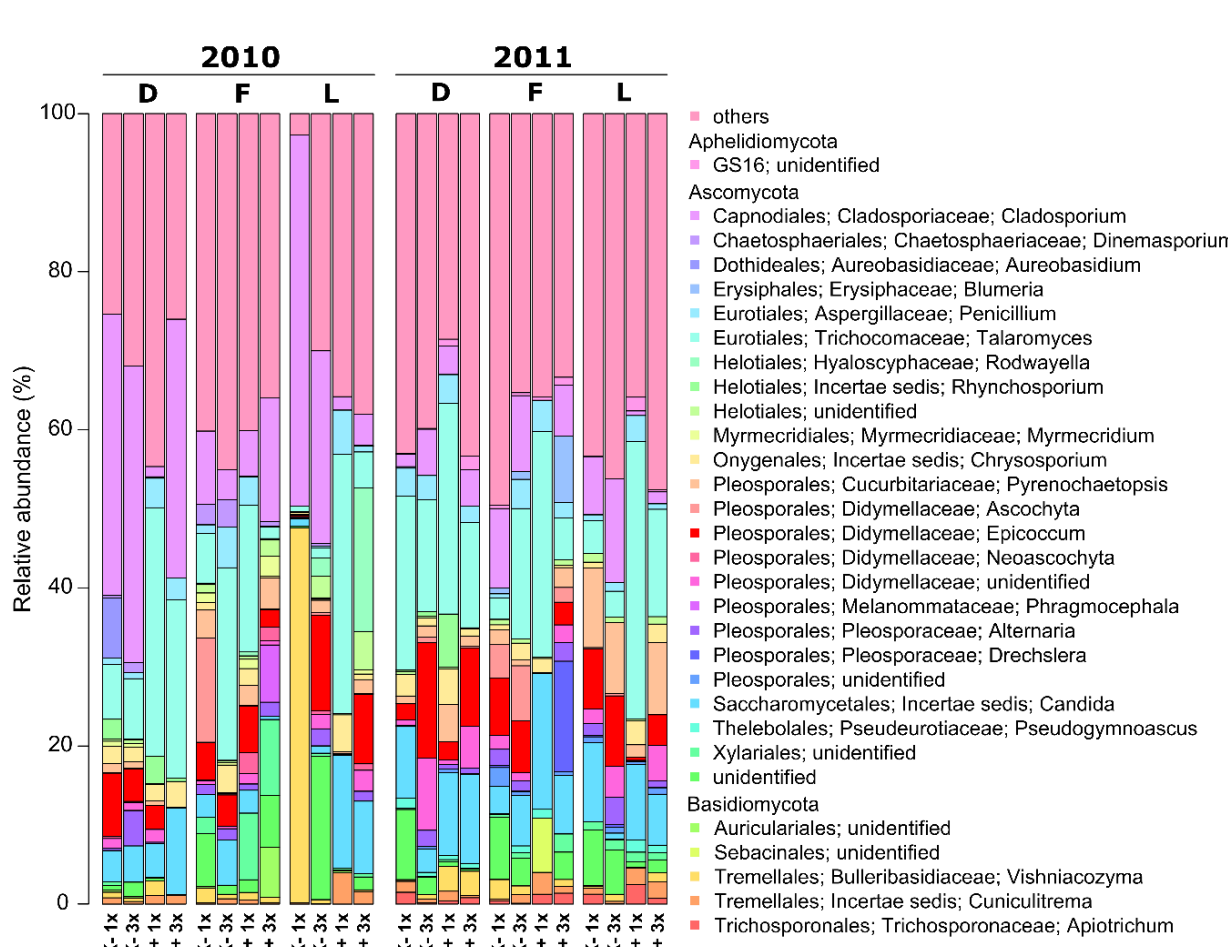
Fungal endophyte community composition at genus level. Fungal endophytes in aerial plant parts of *D. glomerata* (D), *F. rubra* (F) and *L perenne* (L) collected in September 2010 and 2011 were investigated. Three replicates per grass species and treatment were analysed with one exception: *L. perenne* samples were collected only on two non-fertilized plots mown once in 2010. One replicate comprised 10 plants. The relative abundances of the community composition were calculated for each sampling year separately. Only fungal genera with an average abundance >0.5% in the respective year are shown. Abbreviations: NPK-, no fertilizer application; NPK+, with fertilizer application; 1x, mown once a year; 3x, mown three times a year.

Fungi were mainly represented by two phyla: Ascomycota (65.5%) and Basidiomycota (27.7%) (Figure 1, Table S3). The predominant fungal classes within the Ascomycota were Dothideomycetes (27.1%), Eurotiomycetes (18.6%), Saccharomycetes (6.8%), Sordariomycetes (4.6%), and Leotiomycetes (4.5%). The Basidiomycota were dominated by the two classes Agaricomycetes (5.92%) and Tremellomycetes (4.3%). At genus level, *Talaromyces* (14.5%) and *Cladosporium* (11.3%) were predominant. Other abundant fungal genera were, for example, *Candida* (6.6%), *Epicoccum* (5.0%), *Pyrenochaetopsis* (2.5%), *Vishniacozyma* (2.2%), *Penicillium* (2.2%), and *Chrysosporium* (1.8%). However, we detected differences in the relative abundances between the two sampling years. In 2010, *Cladosporium* was the predominant fungal genus in *L. perenne* (16.73%) and *D. glomerata* (26.78%), while *Talaromyces* dominated the fungal community in *F. rubra* in 2010 (12.67%) and 2011 (13.25%)*. Talaromyces* (14.03%) and *Pyrenochaetopsis* (7.44%) were the two predominant fungal genera in *L. perenne* in 2011, while *Talaromyces* (19.03%) and *Candida* (8.46%) dominated the endophyte community of *D. glomerata*.

### 3.2. Sampling Year is a Major Determinant of Fungal Endophyte Communities

To address our hypothesis (i) that fungal endophyte diversity and community composition would differ between the grass species and the two sampling years, we compared fungal diversity (represented by the Shannon diversity index H’), richness (number of observed zOTUs) and phylogenetic diversity (represented by Faith’s PD). In general, *F. rubra* and *D. glomerata* had the highest and the lowest alpha diversity values, respectively (Table 1). However, repeated measures ANOVA revealed only a marginal effect of grass species on fungal richness (F = 2.875; *P* = 0.0638). Sampling year significantly affected fungal diversity (F = 10.6; *P* = 0.002), richness (F = 5.235; *P* = 0.03), and marginally the phylogenetic diversity (F = 3.411; *P* = 0.0694). The management practices and their interaction had no effect on the alpha diversity measures.

The influence of grass species, sampling year and management practices on fungal community profiles was analysed by PERMANOVA. In contrast to alpha diversity measures, grass species had a significant impact on community composition of fungal endophytes (*P* = 0.04). Sampling year also significantly affected endophytic community composition (*P* = 0.03). However, both findings were only true when employing Bray-Curtis dissimilarities. Grass species and sampling year explained 4.48% and 2.82% (Bray-Curtis dissimilarities) and 3.82% and 2.05% (weighted UniFrac dissimilarities) of the variance in the dataset, respectively. We did not observe any direct influence of mowing frequency on fungal community composition in both sampling years (Table 2).

Nonetheless, fertilizer application (*P* = 0.053) and the interaction of mowing frequency with fertilization (*P* = 0.07) had a marginally significant impact on fungal community composition in 2010, but only when using Bray-Curtis dissimilarities. In 2011, we observed a significant effect of fertilizer application and/or the interaction of the two management practices when employing Bray-Curtis and weighted UniFrac dissimilarities. The interaction of mowing frequency with fertilization explained 12.16% or 14.29% (Bray-Curtis dissimilarities) and 10.49% or 18.26% (weighted UniFrac dissimilarities) of the variance in the dataset in 2010 and 2011, respectively.

Most of the predominant genera including *Alternaria*, *Candida*, *Chrysosporium*, *Cladosporium*, *Epicoccum*, *Penicillium*, *Pseudogymnoascus*, *Talaromyces* and *Vishniacozyma* did not differ in their relative abundances with respect to grass species in both sampling years (Figure 1, Table S6). Marginally significant (*P* = 0.052) higher abundances of *Myrmecridium* were recorded in *F. rubra* (1.29%) compared to *D. glomerata* (0.27%) and *L. perenne* (0.18%) in 2010. In addition, *Pyrenochaetopsis* had higher abundances in 2010 samples of *F. rubra* (2.51%) than in *D. glomerata* (0.64%) and *L. perenne* (0.98%). However, this was only marginally significant (*P* ≤ 0.1). In contrast, significantly (*P* = 0.04) higher abundances of this genus were detected in *L. perenne* (7.44%) compared to *D. glomerata* (2.10%) and *F. rubra* (1.28%) in 2011.

Additionally, we performed an indicator species analysis to identify fungal taxa responsible for the observed differences among the grass species (Table 3, Table S4). The lowest and highest number of significantly associated zOTUs was observed for *D. glomerata* and *F. rubra*, respectively. Specifically, two zOTUs affiliated with *Rhynchosporium orthosporum* were significantly associated with *D. glomerata* (average relative abundance in this grass species: 0.52%), whereas zOTUs belonging to *Rodwayella citrinula* (0.85%) and *Pyrenochaetopsis* sp. (1.35%) showed a unique association with *L. perenne*. In addition, several zOTUs affiliated to *Blumeria graminis* (0.63%), *Phragmocephala garethjonesii* (0.18%), *Phomatospora dinemasporium*, (0.18%) and *Ascochyta sorghi* (2.57%) were significantly associated with *F. rubra*.

**Figure 2:**
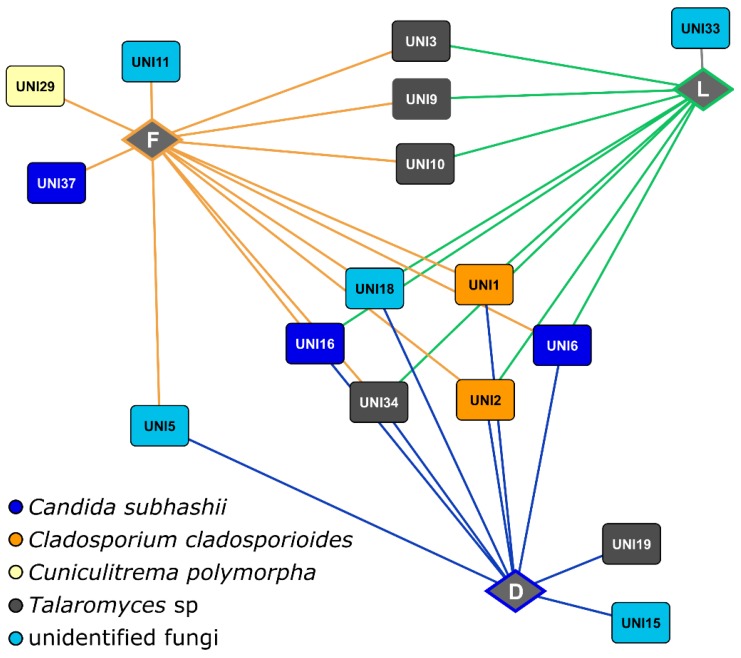
The core community of each grass species as well as fungal zOTUS identified in two or three grass species in both 2010 and 2011. Abbreviations: D, *D. glomerata*; L, *L. perenne*; F, *F. rubra*.

**Figure 3:**
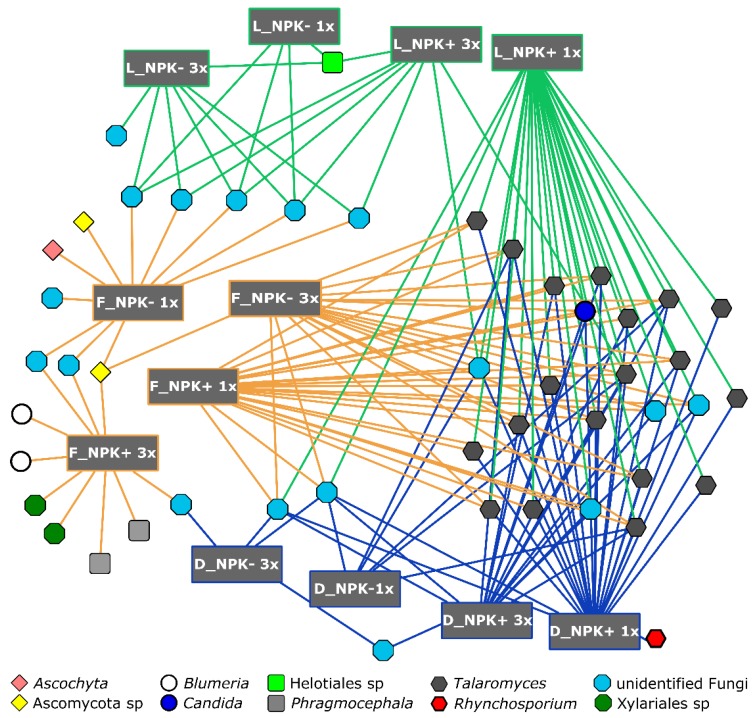
Bipartite association network of fungal zOTUs significantly associated with grass species and management level. Abbreviations: D, *D. glomerata*; L, *L. perenne*; F, *F. rubra*; NPK-, no fertilizer application; NPK+, with fertilizer application; 1x, mown once a year; 3x, mown three times a year.

We were also interested in the core communities of each grass species and if there are fungal zOTUs found in all 71 samples. In total, 16 zOTUs (0.5% of all zOTUS) were identified as core community observed in one, two or three grass species in both sampling years. Although the zOTU number was low, more than 30% of all sequences were assigned to these 16 zOTUs. These zOTUs belonged to *C. cladosporioides*, *Candida subhashii*, *Cuniculitrema polymorpha* and *Talaromyces* sp. (Figure 2). Only a few fungal zOTUs such as zOTUs UNI1, UNI2 or UNI34 were observed in all grass species in both sampling years (Figure 3). The two predominant fungal zOTUs UNI1 (mean relative abundance 5.66%) and UNI2 (mean relative abundance 3.15%) belonged to *C. cladosporioides.* In addition, one zOTU (unidentified fungi) was detected in *L. perenne* plants collected in 2010 and 2011. Four and two zOTUs were found in *F. rubra* and *D. glomerata*, respectively.

### 3.3. Fungal Endophyte Diversity and Community Composition Is Influenced by Management Practices in a Grass Species-Specific Way

We further expected that fungal endophyte communities in the grass species respond differently towards management practices (hypothesis ii). In general, management practices had only limited effects on alpha diversity measures. Nonetheless, a significantly higher phylogenetic diversity of endophytes was observed in *D. glomerata* grown on plots mown three times compared to those mown once (*P* = 0.04) in 2011. In addition, there was a marginally significant effect of management (*P* = 0.09) on the phylogenetic diversity of endophytes in *D. glomerata* in 2011, but not in 2010. Fertilization had a marginally significant impact on fungal richness of *D. glomerata* (*P* = 0.08) and *F. rubra* (*P* = 0.08) in 2011, while fungal richness of *L. perenne* was not affected (*P* = 0.52).

**Figure 4:**
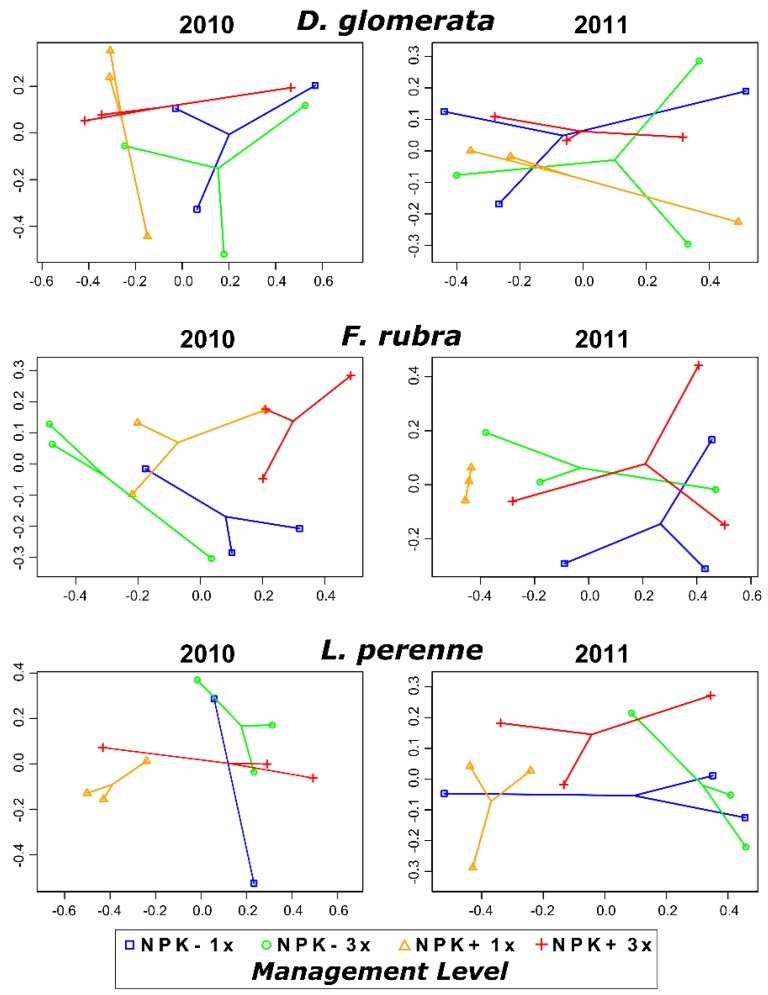
Non-metric multidimensional scaling (nMDS) plots based on Bray-Curtis dissimilarities of fungal endophyte communities in *D. glomerata*, *F. rubra* and *L. perenne* collected in 2010 and 2011. Note that the nMDS axes have different scales for each ordination. Abbreviations: NPK-, no fertilizer application; NPK+, with fertilizer application; 1x, mown once a year; 3x, mown three times a year.

Although not significant, the interaction of grass species and management practices explained more than 36% (Bray-Curtis: *P* = 0.11; weighted UniFrac: *P* = 0.22) and 34% (Bray-Curtis: *P* = 0.15; weighted UniFrac: *P* = 0.37) of the variance in the dataset in 2010 and 2011, respectively. To identify the impact of management practices on fungal community composition in each grass species for each sampling year, we performed non-metric multidimensional scaling (nMDS) analysis for the six subsets. We observed no consistent pattern among the management levels (Figure 4), which is supported by PERMANOVA analysis (Table 2). Neither mowing frequency nor fertilizer application exhibited any significant influence on the community composition in *F. rubra* and *D. glomerata* in both sampling years. 

Conversely, fertilizer application (*P* = 0.047) and the interaction of fertilizer application with mowing frequency (*P* = 0.04) had a significant effect on the fungal community composition of *L. perenne* in 2011 and 2010, respectively, but only when using Bray-Curtis dissimilarities. In addition, the fungal community composition of *L. perenne* was marginally affected by mowing frequency in 2010 (*P* = 0.08; Bray-Curtis dissimilarities) and by fertilizer application in both sampling years (2010: *P* = 0.09; 2011: *P* = 0.08; weighted UniFrac dissimilarities). Although mowing frequency alone did not result in any significant impact on community composition in *L. perenne* in both sampling years, the interaction of this management practice with fertilizer application explained 41.51% or 36.72% (Bray-Curtis dissimilarities) and 41.07% or 38.61% (weighted UniFrac dissimilarities) of the variance in 2010 and 2011, respectively.

### 3.4. The Predominant Fungal Genera Differ in Their Relative Abundances with Respect to Grass Species and Management Regimes 

As we observed contrasting effects of management practices on the grass species, we next compared the relative abundance of the predominant fungal genera between the four management levels for each grass species in 2010 and 2011 (Table S6). Numerous fungal genera differed in their relative abundance in *L. perenne* in 2010 and/or 2011. For example, we recorded significantly higher abundances of *Talaromyces* in *L. perenne* plants collected from fertilized plots mown once a year compared to those from the other management levels in 2010 (*P* < 0.01) and 2011 (*P* < 0.05). Moreover, significantly (*P* < 0.002) higher abundances of *Penicillium* were observed in *L. perenne* samples grown on fertilized plots mown once in 2010. The genera *Alternaria* and *Cladosporium* had significantly (*P* < 0.05) higher abundances in non-fertilized plots mown three times compared to plants collected from fertilized plots 2011. 

In the same year, significantly (*P* < 0.004) higher abundances of *Vishniacozyma* were observed in *L. perenne* samples grown on plots mown three times compared to plants from plots mown once. In addition, we detected higher abundances of *Phragmocephala* in *F. rubra* grown on fertilized plots mown three times compared to all other plots in 2010. However, this was only marginally significant (*P* = 0.06). In 2011, significantly (*P* < 0.03) higher abundances of *Talaromyces* were observed in *F. rubra* grown on fertilized plots mown once compared to non-fertilized plots. In contrast, the relative abundances of the predominant fungal genera in *D. glomerata* plants were not affected by management level in both sampling years.

We further performed an indicator species analysis to identify fungal zOTUs significantly associated with grass species and management level. In total, 160 zOTUs belonging to 10 fungal taxa were identified (Figure 3). The majority of the associated zOTUs (n = 80) was affiliated to the genus *Talaromyces*. The lowest and highest number of significantly associated fungal zOTUs were observed for *F. rubra* (n = 62) and *L. perenne* (n = 45), respectively (Figure 3, Table S4). The number of significantly associated fungal OTUs was higher for *D. glomerata* plants grown on plots mown three times a year than in those mown once a year, and for *F. rubra* and *L. perenne* plants collected from non-fertilized plots mown three times a year.

Only four fungal zOTUs were significantly associated with *D. glomerata* plants grown on fertilized plots and with *L. perenne* plants collected from non-fertilized plots mown once a year. One fungal zOTU (*C. subhashii*) was significantly affiliated with *D. glomerata* grown on fertilised plots mown once and three times. We further identified one zOTU belonging to *A. sorghi* and one zOTU affiliated with *R orthosporum*, which showed unique associations with *F. rubra* grown on non-fertilized plots mown once and with *D. glomerata* grown on fertilized plots mown once a year, respectively. In contrast, two zOTUs affiliated with *B. graminis* and two zOTUs belonging to *P garethjonesii* were associated with *F. rubra* grown on fertilized plots mown three times.

### 3.5. Fungal Functionality Is Only Weakly Affected by Management Level 

**Figure 5:**
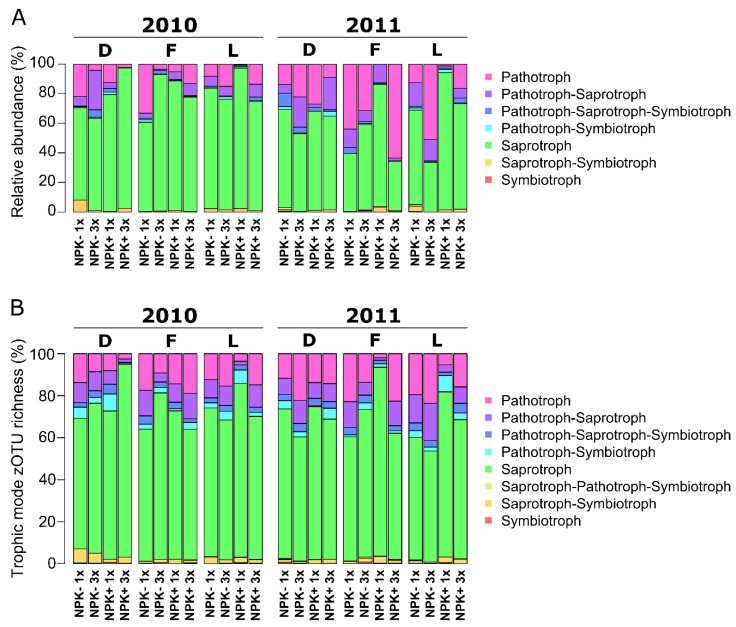
Relative abundance of fungal zOTUs classified by the main trophic modes (**A**) and proportion of zOTU per trophic mode (**B**) in 2010 and 2011. The relative abundances of the trophic modes were calculated for each sampling year separately using the FunGuild database and are based on 31.2% of the zOTUs (n = 1009 zOTUs). Abbreviations: D, *D. glomerata*; L, *L. perenne*; F, *F. rubra*; NPK-, no fertilizer application; NPK+, with fertilizer application; 1x, mown once a year; 3x, mown three times a year.

We hypothesized that the putative life strategies of fungal endophytes are associated with grass species and management practices (hypothesis iii). To test this hypothesis, life strategies (i.e., trophic mode) of endophytic fungi under different management levels were determined using FUNGuild (20). In total, highly-probable and probable life strategies for 1009 of the 3230 zOTUs (= 31.2%) were predicted (Figure 5, Table S5). The current version of FUNGuild supports that fungi can have multiple trophic modes, which are weighted equally (20), resulting in combination of fungal life strategies. Most of the zOTUs were classified as saprotrophs (2010: 78.32%; 2011: 60.27%), while symbiotrophs were the least abundant life strategy in 2010 (0.04%) and 2011 (0.21%) (Figure 5A). We detected five other trophic modes in 2010: pathotrophs (11.17%), pathotroph-saprotrophs (6.42%), pathotroph-saprotroph-symbiotrophs (1.47%), pathotroph-symbiotrophs (0.79%) and saprotroph-symbiotrophs (1.80%). In addition to saprotrophs and symbiotrophs, we found six other trophic modes in 2011: pathotrophs (24.34%), pathotroph-saprotrophs (10.27%), pathotroph-saprotroph-symbiotrophs (2.42%), pathotroph-symbiotrophs (0.94%), saprotroph-pathotroph-symbiotrophs (0.22%), saprotroph-symbiotrophs (1.33%). Similar results were obtained when analysing the zOTU richness (Figure 5B).

The life strategies of the fungal endophytes did not differ in their sequence abundance between the grass species in 2010 and 2011 (Figure 5A, Tables S5 and S7). Nevertheless, the richness of zOTUs assigned as saprotroph-symbiotrophs differed marginally (*P* = 0.08) between *D. glomerata* and in *F. rubra* in 2010, but not in 2011 (Figure 5B). The effect of management level on the putative life strategies of endophytic fungi were subsequently analysed for each grass species and each sampling year separately. We detected no differences (sequence and zOTU richness) in life strategies of *D. glomerata* endophytes among the management levels in 2010 and 2011 and of *F. rubra* and *L. perenne* in 2010. The zOTU richness of saprotrophs in *D. glomerata* plants increased marginally (*P* = 0.054) in plants from fertilized plots mown three times compared to those from non-fertilized plots mown once in 2010. 

We further detected a marginally significant higher fungal zOTU richness of saprotrophs (*P* = 0.052) in *F. rubra* grown on fertilized plots mown once compared to plants from non-fertilized plots mown once and from fertilized plots mown three times in 2011. In the same year, a marginally significant (*P* = 0.052) increase in the relative abundance (sequence richness) of saprotroph-symbiotrophs was observed in *F. rubra* plants from fertilized plots mown once. Moreover, we detected a marginally significant (*P* = 0.051) higher abundance of saprotrophs in *L. perenne* from fertilized plots mown once compared to plants collected from non-fertilized plots mown three times in 2011. 

Interestingly, we detected no differences in the relative abundance of pathotrophs in *F. rubra* and *L. perenne* plants between the management levels in 2010 and in *D. glomerata* for both sampling years (Tables S5 and S7). Nonetheless, a significantly (*P* = 0.03) lower richness of zOTUs assigned as pathotrophs were detected in *F. rubra* grown on fertilized plots mown once than in fertilized plots mown three times and in those from non-fertilized plots mown once in 2011. In the same year, we observed a significantly (*P* = 0.02) higher sequence richness of pathotrops in *L. perenne* grown on non-fertilized plots mown three times than from fertilized plots mown once. However, no differences were detected when investigating the sequence richness.

Overall, we found different responses of fungal endophytes towards fertilizer application and mowing frequency (Table 4). In addition, the observed effects were not consistent among the grass species investigated and differed in both sampling years. 

## 4. Discussion

Fungal endophytes can play an important role for plant growth and health [1,8,10]. However, to date, there are only two comparative studies on fungal endophyte communities in different grass species and both are culture-dependent [14,47]. In the present study, we applied Illumina-based sequencing targeting the fungal ITS2 region to investigate compositional and functional responses of fungal endophyte communities in the agriculturally important grass species *L. perenne*, *D. glomerata* and *F. rubra* over consecutive years. To our knowledge, this is the first study using culture-independent approaches to assess the impact of two management regimes on three agricultural-important grass species.

In line with previous work on fungal endophytes in grass and other plant species [25,48,49], fungal endophyte communities were dominated by Ascomycota. The predominant fungal genera in the present study were *Talaromyces* and *Cladosporium*. Members of the genus *Talaromyces* are well-known for its secondary metabolites, with some having antimicrobial activities [50,51]. The genus *Cladosporium* was mainly represented by zOTUs of the species *C. cladosporioides* and *C. herbarum*. Both species are common, widely distributed fungi, which can be found on dead leaves of numerous plant species or as secondary invader of necrotic plant tissues [52,53]. Wang et al. [54] showed that metabolites from *C. cladosporioides* have antifungal activity against important plant pathogens of the genera *Colletotrichum* and *Phomopsis*. 

The majority of fungal species in this study belonged to Class 3 endophytes, rather than to well-studied clavicipitaceous endophytes (Class 1 endophytes, sensu [55]). In contrast to our study, *Cryptococcus*, *Rhizosphaera*, and *Mycopappus* dominated fungal endophyte communities associated with four vascular plant species [49]. In another study on the effect of cropping systems on fungal endophytes of wheat and faba bean, *Goidanichiella* and *Candida* dominated the aerial plant parts [25]. We postulate that the contrasting results in our and the above-mentioned studies are related to differences in sampling site and/or plant species, as these factors can affect fungal endophyte communities [25,48,56]. 

Fungal alpha diversity measures were not affected by grass species, which is not consistent with previous observations on foliar fungal endophytes of crop plants [25] or tree species [57]. In contrast, no differences in fungal diversity or fungal richness were detected in leaves and roots of two *Agave* species [56], in leaves of two *Acer* species [58] and in stems of the grass species *Triticum dicoccoides* and *Aegilops sharonensis* [48], respectively. In the current study, grass species significantly influenced fungal endophyte community composition. However, we detected no significant influence of grass species when investigating both sampling years separately. This might be related to the high variability among samples from the same species. Similarly, Higgins et al. [59] found no significant differences in fungal endophytes of 11 tropical forest grass species. The authors further showed that no fungal genotype was a significant indicator of any host plant species. This is not in accordance with our study, as several zOTUs were significantly associated with one of the grass species. 

The pathogen *R. orthosporum* was significantly associated with *D. glomerata*, which is known as host grass species of this fungus [60], while *B. graminis* and *A. sorghi* were significantly associated with *F. rubra.* The fungus *B. graminis* includes important, host-specific fungal pathogens of various grasses and cereals of the family Poaceae [61], whereas *A. sorghi* causes leaf spots on numerous grasses and cereals [62]. As we collected healthy plants without any symptoms of disease, this supports the assumption that numerous fungi can live within plants for some time as latent pathogens [5,63,64]. For example, Larran et al. [64] investigated endophytic microorganisms of asymptomatic leaves of *T. aestivum* L. and observed that the most common endophytes in these leaves were wheat pathogens. 

Our study further demonstrated grass species-specific responses of fungal endophytes towards management practices. Previous studies investigating the effect of agricultural practices on foliar fungal communities had contrasting findings [12,18,19,47]. For example, König et al. [12] investigated foliar fungal assemblages of *L. perenne* grown under different land-use intensities (i.e., different mowing, fertilization and grazing regimes) by Illumina-based sequencing and found no effect of land-use intensity on the composition of fungal assemblages. This is partly in line with our study, as the interaction of fertilizer application with mowing frequency had a significant effect on fungal community composition in *L. perenne* in 2010, but not in 2011. A possible explanation for the contrasting finding is that König and colleagues [12] analysed epi- and endophytic fungal assemblages, whereas we investigated endophyte communities. The authors further showed that land-use intensity had no effect on species richness or diversity of fungal assemblages. This is supported by our study, as neither fertilizer application nor mowing frequency affected fungal richness and diversity. 

Contrary to our results for mowing, grazing of reindeer and small rodents promoted the diversity of fungal endophytes in flowering culms of three common tundra grass species [47]. However, we observed a higher phylogenetic fungal diversity in *D. glomerata* plants grown on plots mown three times in 2011. We suggest that frequent mowing promote the growth of distantly related, horizontal transmitted endophytic fungi (Class 3 endophytes, sensu [55]) in aerial plant parts of *D. glomerata*. This resulted in an increasing phylogenetic diversity and, although not significant, higher fungal richness. According to Ernst and colleagues [65], niche differentiation is a major determinant of fungal endophyte communities. This might play a role in the present study: compared to *L. perenne* and *F. rubra*, *D. glomerata* as a tall-growing grass species and thus provides slightly different niches than the other two grass species investigated.

A possible explanation for the contrasting findings in our and the above-mentioned studies is that fungal endophytes from different grass species and/or genotypes from different regions were investigated, suggesting that the responses of these communities towards management practices might differ. Another explanation is that the grass species select for beneficial microorganisms as they provide a selective advantage for their host plant to better adapt to environmental constraints as suggested by Vandenkoornhuyse and colleagues [66]. These assumptions are partly confirmed by our correlation-based indicator species analysis. We identified several fungal zOTUs with putative plant growth and health-promoting potential, such as *C. subhashii* or several *Talaromyces* species, which were significantly associated with one grass species and one management level. For example, Hilber-Bodmer et al. [67] showed recently that the yeast *C. subhashii* strongly antagonized a wide range of filamentous fungi. In addition, we identified several pathogenic fungi such as *R. orthosporum*, *B graminis* and *A. sorghi* [60,61,62]. However, we can only speculate that the above-mentioned fungal species have plant growth/health-promoting or pathogenic abilities as fungal species are able to switch in their life style based on the host plant [5,68]. In addition, we cannot determine if the grass species selected for beneficial fungi or if there are other forces which resulted in host specificity.

Interestingly, we found only a few zOTUs as core community in one or two of the grass species investigated. Moreover, two abundant zOTUs belonging to *C. cladosporioides* were present in all plant samples. These findings suggest that these fungi are seed-borne. This is supported by a previous study on seed fungal endophytes of the coastal plant *Suaeda salsa* assessed by pyrotag-based sequencing [69]. Here, the genus Cladosporium dominated the seeds. The authors further showed that *C. cladosporioides* significantly promoted host seed germination and plant growth, which might play a role in the present study. The authors stated that the transmission mode of *C. cladosporioides* in *S. salsa* remains elusive. In a previous study on endophytes from six forb species, the two endophyte species *Alternaria alternata* and *C. sphaerospermum* were vertically transmitted [70]. Vertical transmission of pathogenic [71,72] and beneficial endophytic fungi such as *Neotyphodium* [55,73] is well-known. However, as we did not investigate seedling or seed endophytes, we cannot fully determine if the fungi observed as core community in the grass species or found in all samples were horizontally or vertically transmitted. Consequently, future studies should analyse fungal endophytes from seeds and seedlings as well.

In the present study, the effects of grassland management practices on endophyte communities differed not only among the grass species, but also between the sampling years. This is in line with previous studies showing that sampling time influenced fungal endophyte communities [56,59]. We speculate that the grass species were colonized by different horizontal transmitted endophytic fungi (Class 3 endophytes) in both sampling years. This is supported by the low number of zOTUS observed as core community in each grass species. The interaction of grass species and management practices explained only 36% and 34% of the variance in the dataset in 2010 and 2011, respectively, indicating that the fungal communities were affected by other factors not determined in the present study.

We observed differences between the two dissimilarities used, as significant effects were mainly detected when using Bray-Curtis dissimilarities. Bray-Curtis dissimilarities are taxonomy-independent, whereas UniFrac dissimilarities incorporate the phylogenetic relationships between zOTUs. Our results indicate that fungal endophyte communities were taxonomically related to each other, i.e., different but closely related fungal species colonized the grass species in the two sampling years, and that closely related taxa are potentially ecologically equivalent. Moreover, these results highlight that the use of two different dissimilarities for beta diversity analysis can give an additional insight into fungal community composition. 

We applied FUNGuild [23] to better understand functional responses of fungal endophytes towards grass species and management practices. In line with a recent study investigating root-associated fungal communities in the Bolivian Andes [74], saprophytic fungi dominated endophyte communities, while symbiotrophs were the least abundant life strategy. The high abundance of saprophytic fungi might be related to the late sampling time in autumn, as several endophytic fungi can shift their life strategy to saprophytic when the host senesces [75,76]). A possible explanation is given by Porras-Alfaro and Bayman [5]: saprotrophs that can colonize living plant tissues are able to start growing as soon as the host tissue senesces, which give them a competitive advantage over other saprotrophs that colonize plants later. 

Contrary to changes in community composition, the ecological life strategies did not differ among the grass species. However, we observed differences when analysing the sequence richness (number of zOTUs assigned to a specific guild divided by the number of all assigned zOTUs) and zOTU richness (the proportion of zOTUs assigned to a specific trophic mode), supporting the suggestion of Nguyen et al. [23] that combining both dimensions reflect the relative importance of fungal life strategies in a particular environment. The low number of zOTUs (31.2%) used in the analysis indicates that the role of most fungi in the plant endosphere and their ecological roles remain largely unknown, as proposed by Rodriguez et al. [55].

## 5. Conclusions

In the current study, we showed that sampling year and grass species had a significant impact on fungal endophyte community composition. In addition, sampling year significantly affected fungal diversity and richness but not the phylogenetic diversity. These results are only partly in line with our first hypothesis that fungal endophyte diversity and community composition differ among the investigated grass species and between the sampling years. We further observed grass species-specific responses of fungal endophytes towards management regimes, which supports our second hypothesis. However, the interaction of grass species and management practices explained only 36% and 34% of the variance in the dataset in 2010 and 2011, respectively, indicating that other factors than those investigated are important drivers of fungal endophyte communities in the grass species analysed in this study. Contrary to our third hypothesis that putative life strategies of fungal endophytes are determined by grass species and management practices, grass species had no effect on the fungal life strategies. However, we observed grass species-specific responses of the fungal life strategies towards management practices, which were not consistent in both sampling years. We found a few zOTUs, which were present in all plant samples, suggesting that these fungi were vertically transmitted. We suggest that future studies should include endophytes from seeds and grass seedlings to identify vertically transmitted fungi. In addition, the number of grass species should be extended to get a more comprehensive picture on how fungal endophytes respond to different management practices. Nonetheless, our results provide a basis for further studies on responses of fungal endophytes and their life strategies in agriculturally important grass species towards management practices.

## Figures and Tables

**Table 1:** Alpha diversity of fungal endophyte communities in *D. glomerata*, *F. rubra* and *L. perenne* under different management levels in 2010 and 2011.

	Richness (Number of Observed zOTUs)		Diversity (Shannon Diversity Index H’)		Phylogenetic Diversity (Faith’s PD)	
	2010	2011	2010	2011	2010	2011
***D. glomerata***	402 ± 219	499 ± 80	3.71 ± 1.66	4.63 ± 0.38	62.98 ± 28.96	70.44 ± 6.54
***F. rubra***	531 ± 93	565 ± 112	4.66 ± 0.4	4.82 ± 0.46	75.79 ± 14.88	80 ± 15.98
***L. perenne***	442 ± 224	541 ± 96	3.72 ± 1.69	4.86 ± 0.25	66.98 ± 21.92	79.59 ± 14.21
***D. glomerata***						
NPK- 1x	309 ± 262	337 ± 66	3.44 ± 1.83	4.05 ± 0.36	26.55 ± 19.29	25.22 ± 0.68
NPK- 3x	287 ± 230	423 ± 49	3.22 ± 2.21	4.41 ± 0.09	25.98 ± 17.15	27.16 ± 5
NPK+ 1x	421 ± 44	392 ± 22	4.29 ± 0.15	4.4 ± 0.09	27.14 ± 6.57	24.4 ± 2.22
NPK+ 3x	258 ± 160	444 ± 57	3.15 ± 1.93	4.49 ± 0.22	19.67 ± 6.91	27.51 ± 2.21
***F. rubra***						
NPK- 1x	478 ± 90	512 ± 52	4.57 ± 0.5	4.85 ± 0.4	30.65 ± 5.43	34.97 ± 6.17
NPK- 3x	363 ± 84	516 ± 122	4.18 ± 0.32	4.74 ± 0.44	26.03 ± 3.06	32.96 ± 6.36
NPK+ 1x	469 ± 39	362 ± 53	4.58 ± 0.26	4.21 ± 0.28	32.59 ± 4.25	23.2 ± 1.33
NPK+ 3x	463 ± 60	411 ± 71	4.43 ± 0.53	4.35 ± 0.4	34.63 ± 6.14	32.01 ± 9.22
***L. perenne***						
NPK- 1x	121 ± 1	419 ± 117	1.08 ± 0.07	4.44 ± 0.4	17.2 ± 0.6	31.54 ± 5.09
NPK- 3x	401 ± 260	484 ± 27	3.75 ± 1.86	4.76 ± 0.06	28.36 ± 15.66	32.67 ± 2.62
NPK+ 1x	440 ± 73	434 ± 156	4.54 ± 0.19	4.58 ± 0.43	30.16 ± 5.69	32.16 ± 11.6
NPK+ 3x	378 ± 154	454 ± 60	3.9 ± 0.99	4.71 ± 0.15	27.72 ± 6.71	32.24 ± 5.91

Abbreviations: NPK-, no fertilizer application; NPK+, with fertilizer application; 1x, mown once a year; 3x, mown three times a year.

**Table 2:** Effect of grass species and grassland management regimes on fungal endophyte community composition in 2010 and 2011 based on Bray-Curtis and weighted UniFrac dissimilarities.

	2010				2011			
	Bray-Curtis		Weighted UniFrac		Bray-Curtis		Weighted UniFrac	
All Grass Species	*R^2^ (%)*	*P*	*R^2^ (%)*	*P*	*R^2^ (%)*	*P*	*R^2^ (%)*	*P*
Grass species (G)	6.75	0.25	8.00	0.19	7.55	0.15	3.86	0.61
Mowing frequency (M)	2.52	0.56	1.62	0.748	4.27	0.14	5.81	0.12
Fertilizer application (F)	5.16	0.053	5.14	0.133	6.17	**0.05**	8.53	**0.048**
Fertilization * Mowing	12.16	0.07	10.49	0.271	14.29	**0.04**	18.26	0.053
G * F * M	36.69	0.11	36.89	0.22	35.96	0.15	34.05	0.37
***D. glomerata***								
Mowing frequency	5.07	0.86	1.77	0.92	7.95	0.37	6.83	0.43
Fertilizer application	11.33	0.26	13.26	0.18	4.62	0.91	3.31	0.73
Fertilization * Mowing	23.71	0.64	17.32	0.76	17.68	0.92	13.47	0.88
***F. rubra***								
Mowing frequency	6.36	0.73	2.98	0.92	6.36	0.64	4.58	0.60
Fertilizer application	10.74	0.28	6.59	0.50	11.69	0.21	12.77	0.22
Fertilization * Mowing	30.74	0.25	30.27	0.37	38.86	0.11	47.00	0.10
***L. perenne***								
Mowing frequency	16.23	0.08	14.89	0.14	10.36	0.26	12.65	0.23
Fertilizer application	17.22	0.054	17.38	0.09	20.56	**0.047**	21.92	0.08
Fertilization * Mowing	41.51	**0.04**	41.07	0.10	36.72	0.16	38.61	0.22

Significant (*P*  ≤  0.05) and marginally significant (*P*  ≤  0.10) p values are written in bold and are underlined, respectively.

**Table 3:** Significantly associated fungal zOTUs with each grass species. The name of the zOTUs refers to the OTU-ID (see Table S2). The relative abundance of each zOTU in the entire dataset (2010 and 2011) as well as for their respective host grass species are shown.

		Relative Abundance (%)	
Fungal Species	zOTU (OTU-ID)	Entire Dataset	Grass Species
***D. glomerata* (n = 9)**			
*Rhynchosporium orthosporum*	UNI153	0.10	0.31
	UNI243	0.07	0.21
Fungi sp.	UNI25	0.76	2.04
	UNI41	0.48	0.48
	UNI68	0.35	0.90
	UNI92	0.23	0.65
	UNI136	0.17	0.42
	UNI240	0.08	0.20
	UNI967	0.09	0.22
***F. rubra* (n = 21)**			
*Ascochyta sorghi*	UNI32	0.54	1.53
	UNI76	0.25	0.72
	UNI175	0.11	0.32
*Phragmocephala garethjonesii*	UNI223	0.06	0.18
*Blumeria graminis*	UNI130	0.15	0.42
	UNI231	0.07	0.21
*Myrmecridium* sp	UNI187	0.16	0.34
*Phomatospora dinemasporium*	UNI145	0.09	0.18
Xylariales sp.	UNI128	0.14	0.23
	UNI131	0.14	0.32
	UNI222	0.08	0.11
	UNI251	0.06	0.12
Ascomycota sp.	UNI93	0.21	0.48
	UNI114	0.12	0.30
Fungi sp.	UNI13	1.11	2.34
	UNI35	0.51	1.0
	UNI60	0.25	0.68
	UNI75	0.25	0.68
	UNI89	0.28	0.56
	UNI106	0.13	0.35
	UNI141	0.15	0.29
***L. perenne* (n = 14)**			
*Pyrenochaetopsis* sp	UNI44	0.34	0.60
	UNI78	0.23	0.42
	UNI97	0.17	0.33
*Rodwayella citrinula*	UNI69	0.20	0.58
	UNI126	0.09	0.27
Helotiales sp.	UNI203	0.07	0.15
Fungi sp.	UNI14	0.80	1.5
	UNI24	0.53	1.07
	UNI39	0.27	0.5
	UNI72	0.18	0.36
	UNI149	0.24	0.45
	UNI167	0.15	0.3
	UNI173	0.15	0.28
	UNI236	0.08	0.2

**Table 4:** Summary of findings. Effect of mowing frequency and fertilizer application on fungal endophyte communities of *D. glomerata*, *F. rubra* and *L. perenne* in 2010 and 2011. The putative life strategies are represented as sequence richness (number of zOTUs assigned to a specific guild divided by the number of all assigned zOTUs) and zOTU richness (the proportion of zOTUs assigned to a specific trophic mode).

	*D. glomerata*		*F. rubra*		*L. perenne*	
	2010	2011	2010	2011	2010	2011
**Richness (number of observed zOTUs)**						
**Mowing (M)**	**-**	**-**	**-**	**-**	**-**	**-**
**Fertilization (F)**	**-**	*	**-**	*	**-**	**-**
F * M	**-**	**-**	**-**	**-**	**-**	**-**
**Diversity (Shannon diversity index H’)**						
**Mowing (M)**	**-**	**-**	**-**	**-**	**-**	**-**
**Fertilization (F)**	**-**	**-**	**-**	**-**	**-**	**-**
F * M	**-**	**-**	**-**	**-**	**-**	**-**
**Phylogenetic diversity (Faith’s PD)**						
**Mowing (M)**	**-**	**+**	**-**	**-**	**-**	**-**
**Fertilization (F)**	**-**	**-**	**-**	**-**	**-**	**-**
F * M	**-**	*	**-**	**-**	**-**	**-**
**Community composition (Bray-Curtis/ weighted UniFrac dissimilarities)**						
**Mowing (M)**	**-/-**	**-/-**	**-/-**	**-/-**	*/-	**-/-**
**Fertilization (F)**	**-/-**	**-/-**	**-/-**	**-/-**	*/*	+/*
F * M	**-/-**	**-/-**	**-/-**	**-/-**	+/*	**-/-**
**Sequence richness**						
F * M	**-**	**-**	**-**	*	**-**	+/*
				*Saprotroph-* *Symbiotroph*		*Pathotroph/ Saprotroph*
**zOTU richness**						
F * M	*	**-**	**-**	+/*	*	*
				*Pathotroph/* *Pathotroph-* *Saprotroph, Saprotroph, Saprotroph-* *Symbiotroph*		*Pathotroph-* *Symbiotroph*

+, significant (*P* ≤ 0.05); -, not significant (*P* > 0.05); *, marginally significant (*P* ≤ 0.1).

## Supplementary Materials

The following are available online at http://www.mdpi.com/2076-2607/7/2/37/s1, Figure S1: Rarefaction (A) and species accumulation (B) curves., Table S1: Sample characteristics. Note that sample L47_S2011 was sequenced twice (SeqIDs BEWE_a_699 and BEWE_a_719). Table S2: zOTU table for the fungal endophyte community. For further information on the Sample ID see Supplementary Table S1. Table S3: Sequence characteristics (number of sequences) and alpha diversity values of the fungal dataset. Table S4: Significantly (p < 0.05) associated fungal zOTUs with grass species and management practices. Table S5: Putative life strategies of fungal endophytes in 2011. Table S6: Differences in the relative abundances of fungal endophytes between grass species tested by pairwise comparison. Table S7: Differences in the putative life strategies of fungal endophytes between grass species tested by pairwise comparison.
