# Supplementary figures and images for: Fungal Endophyte Communities of Three Agricultural Important Grass Species Differ in Their Response Towards Management Regimes
Source: Microorganisms. 2019 Jan 27;7(2):37. doi: 10.3390/microorganisms7020037 (PMC6407066; doi:10.3390/microorganisms7020037)

(a)

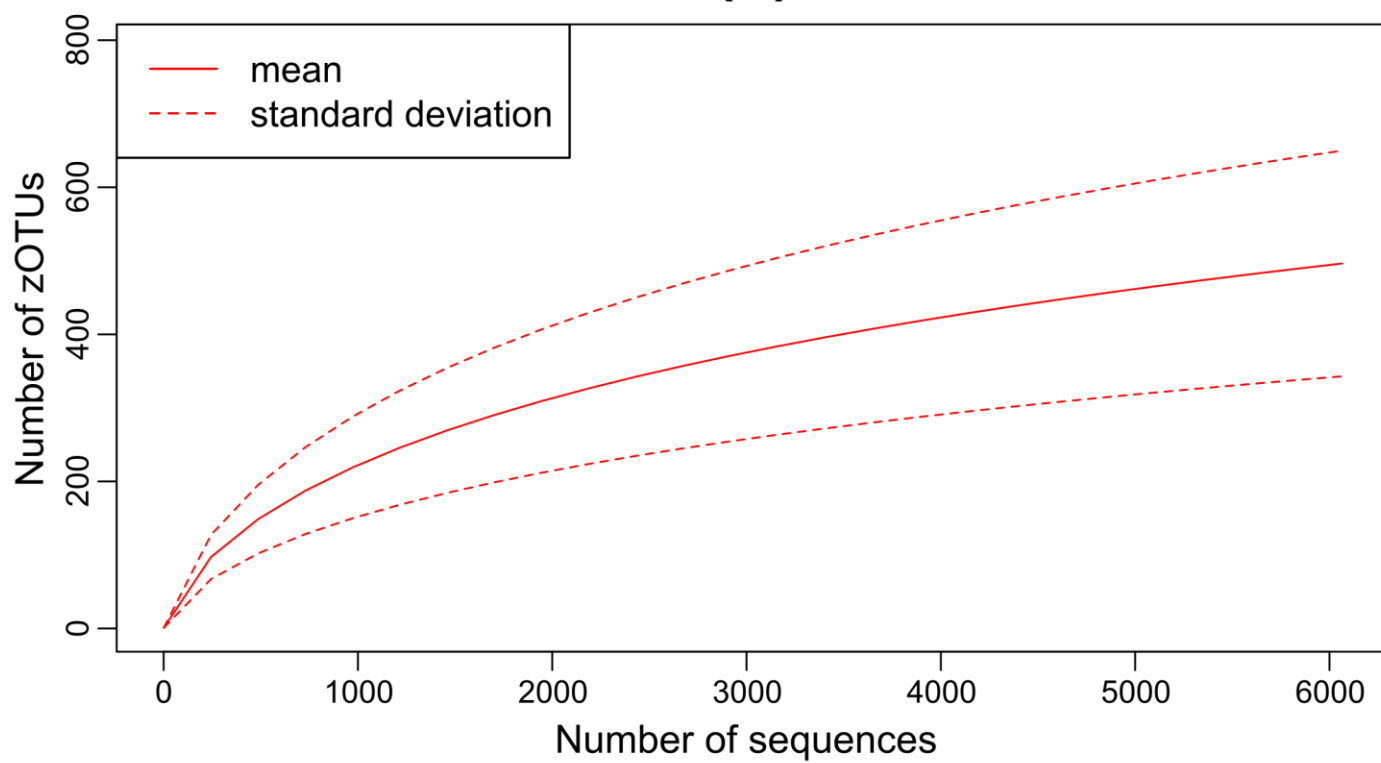

(b)

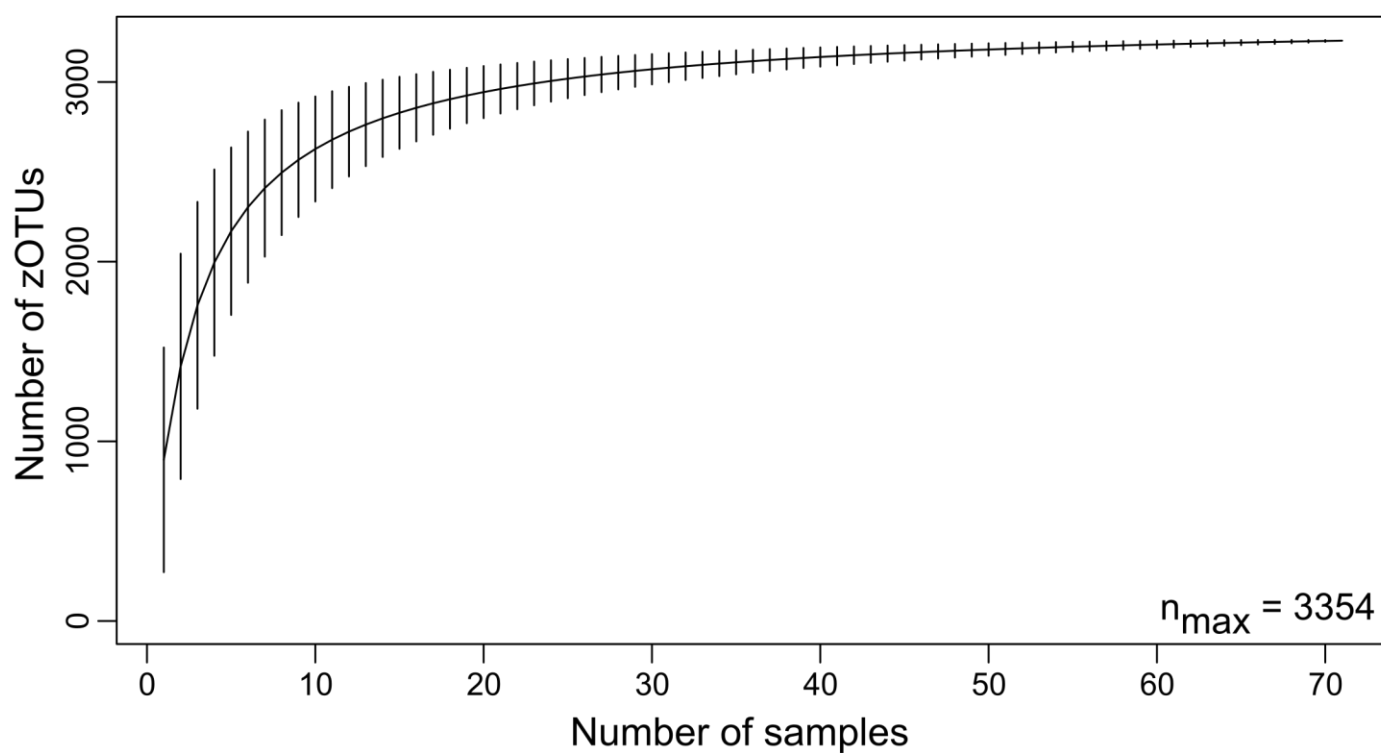

**Supplementary Figure S1.** Rarefaction (A) and species accumulation (B) curves.

Supplement: Supplementary file 1 [file microorganisms-07-00037-s001.zip › FigureS1.pdf]
